# Causal effect between chronic periodontitis and IgA nephropathy: Insights from a 2-sample bidirectional Mendelian randomization study

**DOI:** 10.1097/MD.0000000000049419

**Published:** 2026-06-19

**Authors:** He Miao, Feifei Guan, Jiaguo Huang, Runmiao Hua

**Affiliations:** a Department of Urology, The First People’s Hospital of Xiaoshan, Hangzhou, Zhejiang, China; b Physical Examination Center, Affiliated Xiaoshan Hospital, Hangzhou Normal University, Hangzhou, Zhejiang, China; c Department of Urology, Affiliated Xiaoshan Hospital, Hangzhou Normal University, Hangzhou, Zhejiang, China.

**Keywords:** causality, chronic periodontitis, genome-wide association studies, IgA nephropathy, Mendelian randomization

## Abstract

Epidemiological evidence suggests an association between chronic periodontitis and immunoglobulin A nephropathy (IgAN), although this association remains controversial. This study aimed to investigate the causal effect between chronic periodontitis and IgAN using a 2-sample bidirectional Mendelian randomization (MR) analysis, using single nucleotide polymorphisms as genetic instrumental variables obtained from publicly available genome-wide association study data. The main MR analysis employed the inverse variance weighted method, complemented by supplementary analyses utilizing MR-Egger and weighted median methods. Heterogeneity was analyzed using the Cochran *Q* test, and pleiotropy was evaluated using the MR-Egger intercept test. A leave-one-out analysis was used to identify potentially influential single nucleotide polymorphisms, and the MR-pleiotropy residual sum and outlier method method was used to identify outliers. The inverse variance weighted results indicated that there was no genetic evidence for a strong causal effect of chronic periodontitis on IgAN in the genetic prediction (odds ratio = 1.02, 95% confidence interval = 0.97–1.07, *P* = .42). There was no genetic evidence for a strong causal effect of IgAN on chronic periodontitis (odds ratio = 1.19, 95% confidence interval: 0.92–1.53, *P* = .18). This MR study found no genetic evidence for a strong causal effect between chronic periodontitis and IgAN in the European population.

## 1. Introduction

Immunoglobulin A nephropathy (IgAN) is considered the most common primary glomerulonephritis globally and a common etiological factor for end-stage renal disease.^[1,2]^ The prevalence of IgAN differs across regions and is higher among Asian populations compared to White populations.^[3]^ Moreover, IgAN is more prevalent among young adults,^[4]^ and end-stage renal disease occurs in 20 to 40% of patients within 10 to 20 years.^[5]^ Furthermore, IgAN is a chronic condition that lasts a lifetime, and its severe symptoms and manifestations are strongly associated with a poor prognosis, placing significant psychological and financial burdens on patients. Currently, the etiology and pathogenesis of IgAN remain incompletely understood. It is hypothesized that the pathogenesis of IgAN involves mucosal immunity, in which dysbiosis of the gut microbiota and the subsequent immune responses to commensal flora are pivotal in disease initiation and progression.^[6–9]^

Chronic periodontitis is a chronic multifactorial inflammatory condition primarily driven by oral anaerobic bacteria resulting from a combination of genetic, immunological, environmental, microbial, and lifestyle factors.^[10]^ Periodontitis affects approximately 45 to 50% of the population, and severe periodontitis affects 11.2% of the population, making it the sixth most prevalent disease.^[11–13]^ Previous studies have displayed a link between periodontitis and IgAN.^[14,15]^ Patients with IgAN had a higher prevalence of chronic and invasive periodontitis than those without IgAN, suggesting a strong link between IgAN and chronic periodontitis.^[16,17]^ In the Xinjiang Uygur Autonomous Region of China, the prevalence of IgAN among children with periodontal disease is as high as 53%, far exceeding the 29% observed in patients without periodontal disease.^[18]^ Previous epidemiological studies are undoubtedly affected by various confounding factors and reverse causality bias. Therefore, there is still no consensus on whether chronic periodontitis leads to IgAN.

Mendelian randomization (MR) is an epidemiological study design that limits confounding bias and reverse causality bias compared with observational studies. In MR analysis, genetic variants firmly linked to a modifiable exposure are employed as genetic instrumental variables (IVs) to determine the causal relationship between the exposure and outcome.^[19,20]^ With the exponential expansion and wide application of genetic data, MR is extensively utilized in various diseases. This study aimed to use genetic data to investigate the causal effect between chronic periodontitis and IgAN through a 2-sample bidirectional MR analysis.

## 2. Methods

No additional ethical approval was necessary because the data were obtained from publicly accessible databases that had previously been granted ethical approval and informed consent.

**Figure 1.**
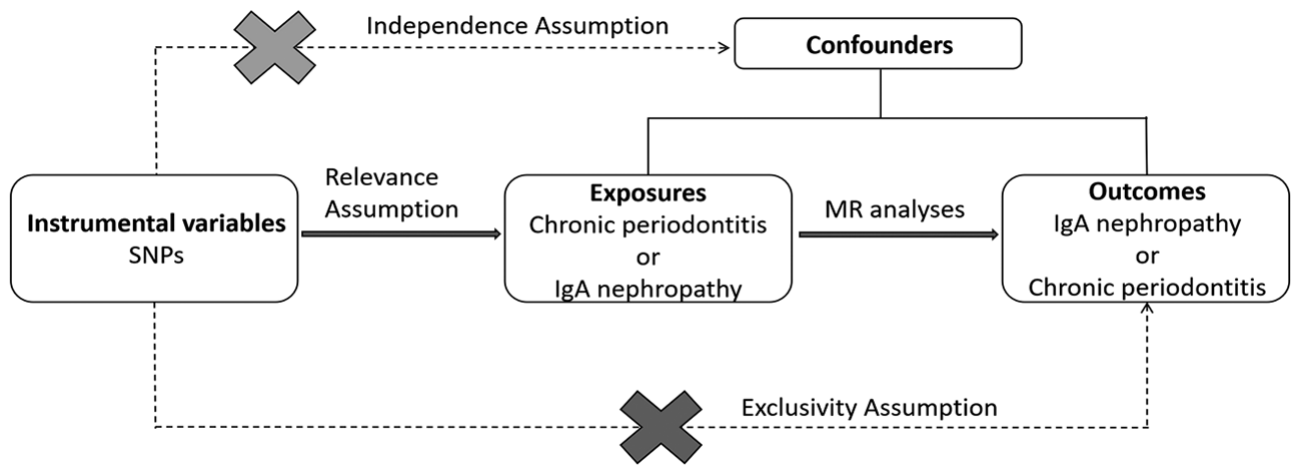
The overview of the study design. IgA = immunoglobulin A, MR = Mendelian randomization, SNP = single nucleotide polymorphism.

This study aimed to investigate the causal effect between chronic periodontitis and IgAN through a 2-sample bidirectional MR analysis, utilizing single nucleotide polymorphisms (SNPs) as genetic IVs. This method requires the fulfillment of 3 assumptions: SNPs must be strongly related to exposure; SNPs should not have confounding factors that could affect the causal relationship between exposure and outcome; SNPs should only influence the outcome through the exposure, without involving any other means.^[19]^ The study design is illustrated in Figure 1.

The genetic instruments were sourced from genome-wide association studies (GWAS) through MR-base.^[21]^ We identified SNPs associated with chronic periodontitis and IgAN at a genome-wide significance level (*P* < 5 × 10^−6^) with rigorous pairwise linkage disequilibrium *R*^2^ < 0.001 from GWAS summary data.^[22]^ In the European population, the GWAS summary data for chronic periodontitis, sourced from the FinnGen project (OpenGWAS ID: finn-b-K11_PERIODON_CHRON), comprised 198,441 individuals (3046 cases and 195,395 controls). The Finnish adaptation of the International Classification of Diseases codes served as the basis for the definition of chronic periodontitis. Additionally, GWAS summary data for IgAN (OpenGWAS ID: ebi-a-GCST90018866) consisted of 477,784 individuals, including 15,587 cases and 462,197 controls. These data for IgAN (International Classification of Diseases , Tenth Revision: N02.8, phecode: 593) were sourced from a meta-analysis combining GWAS data from the United Kingdom Biobank (sample size = 344,365) and the FinnGen project (sample size = 133,419), and were stored in the IEU database.^[23]^ The authors did not gather any identifying information about individual participants during or after data collection. Palindromic SNPs and SNPs with incompatible alleles, such as adenine (A)/guanine (G) and A/cytosine (C), were excluded from this study. Subsequently, we verified these SNPs in the LDtrait Tool (https://ldlink.nci.nih.gov/?tab=ldtrait) to ensure they were not associated with any confounding factors. Additionally, *R*^2^ and *F* values were calculated for each SNP to assess the statistical strength of IVs to prevent bias from weak IVs.^[24]^ The formulae are given below.


$$R^{2}=\frac{2\times \left(1-EAF\right)\times \left(EAF\right)\times \beta^{2}}{2\times \left(1-EAF\right)\times \left(EAF\right)\times \beta^{2}+2\times \left(1-EAF\right)\times \left(EAF\right)\times N\times SE^{2}}$$

$$\begin{array}{c} F=\;\;\;\;\;\frac{R^{2}\times \left(N-2\right)}{1-R^{2}} \end{array}$$

Note: β, effect size; k, number of SNPs; EAF, effect allele frequency; N, sample size; SE, standard error.

All MR analyses were conducted using the packages TwoSampleMR (version 0.6.21) and MR pleiotropy residual sum and outlier method (MR-PRESSO) (version 1.0) in R (version 4.5.1; R Foundation ). Following the harmonization of the effect alleles across the GWAS of chronic periodontitis and IgAN, we employed several MR methods to determine MR estimates between chronic periodontitis and IgAN, including inverse variance weighted (IVW), weighted median, and MR-Egger. Multiple methods were used because of their different underlying assumptions for horizontal pleiotropy. The primary outcome was derived from an IVW meta-analysis of the Wald ratio for each SNP under the assumption that the instruments affect the outcome exclusively through the exposure of interest without alternative pathways.^[25]^ MR-Egger and weighted median methods were also employed to complement IVW estimates because they can provide more reliable estimates across diverse scenarios, albeit with less efficiency, resulting in a wider confidence interval (CI). In cases of inconsistent estimates from these methods, a tighter *P* value threshold for the instrument was applied.^[26]^

In MR studies, sensitivity analysis plays a vital role in identifying underlying pleiotropy and can significantly compromise the heterogeneity of MR estimates. Heterogeneity markers (Cochran *Q* test derived *P* < .05) from the IVW method were used to indicate potential horizontal pleiotropy. The intercept obtained from the MR-Egger regression served as a marker for directional pleiotropy, with *P* < .05 indicating its presence.^[27]^ To evaluate and adjust for horizontal pleiotropy, the MR-PRESSO was used.^[26]^ It demonstrates less bias and greater accuracy compared to IVW and MR-Egger when horizontal pleiotropy variants are < 10%.^[28]^ A leave-one-out analysis was also used to determine whether MR estimates were affected or biased by any individual SNP. To mitigate the winner’s curse bias, we employed the MRlap package to adjust the IVW results. This method accounts for both weak instrument bias and the winner’s curse while also considering potential sample overlap.^[29,30]^ This method helps minimize these biases in the analysis. The power calculations were conducted using an online platform (https://shiny.cnsgenomics.com/mRnd/).^[31]^

## 3. Results

This study conducted a 2-sample bidirectional MR analysis to explore the association between chronic periodontitis and IgAN. A total of 16 SNPs were identified as being significantly associated with chronic periodontitis, and 27 SNPs were identified as being significantly associated with IgAN (*P* < 5 × 10^−6^, LD *R*^2^ < 0.001). The LDtrait Tool was used to eliminate confounding factors. To ensure that the effect was aligned with the same allele, 9 independent SNPs associated with chronic periodontitis and 11 independent SNPs associated with IgAN were selected for the causal effect assessment, with the outcome data harmonized accordingly. Additionally, allele information was compared to confirm that these SNPs were not palindromic. Among all SNPs, *F* statistic values exceeded the conventional threshold of 10 (*F* = 20.91–22.74, *P* < .001), suggesting strong potential for these instruments. The details of these SNPs are listed in Table 1.

**Table 1:** Detailed information on SNPs for MR analysis when chronic periodontitis and IgA nephropathy are exposure.

Phenotype	Position	SNP	Effect allele	Other allele	EAF	Beta	SE	*P* value	*R* ^2^	*F*
Chronic periodontitis	31,826,100	rs112573355	C	G	0.003382	1.2283	0.2576	1.85802E−06	0.000114561	22.73593449
	122,458,770	rs115399664	T	C	0.008944	0.6732	0.1464	4.26098E−06	0.000106544	21.14470761
	4,251,861	rs56222175	T	A	0.03672	0.3368	0.0717	2.61198E−06	0.00011118	22.06487529
	32,604,769	rs61427077	G	C	0.2743	−0.1371	0.0296	3.55001E−06	0.000108097	21.45295444
	49,250,265	rs73404204	G	A	0.02504	0.3962	0.0861	4.22299E−06	0.000106695	21.17474858
	145,696,377	rs75175128	G	A	0.02503	0.3928	0.0859	4.74898E−06	0.000105361	20.90991433
	188,604,115	rs7690117	C	G	0.3013	0.1339	0.0284	2.50697E−06	0.000112007	22.22900875
	163,913,563	rs77930255	T	C	0.01675	0.4767	0.1036	4.21998E−06	0.000106682	21.17222087
	15,012,390	rs9648195	A	C	0.2312	0.145	0.0312	3.37101E−06	0.00010883	21.59844274
IgAN	89,035,280	rs140069999	A	G	0.0496837	−0.2748	0.0471	5.292E−09	7.12409E−05	34.04002141
	6,774,485	rs143129886	C	T	0.0135229	−0.3128	0.0671	0.000003155	4.54817E−05	21.7313462
	159,830,547	rs145091230	C	T	0.00645182	0.3303	0.069	1.70498E−06	4.79586E−05	22.91485682
	114,180,688	rs17184837	T	G	0.0284452	0.1756	0.0354	7.28803E−07	5.14978E−05	24.60598082
	126,932,902	rs1927355	T	C	0.393503	0.0624	0.0118	1.28499E−07	5.85259E−05	27.96426099
	127,438,484	rs2885134	G	A	0.0243264	0.2128	0.0358	2.73602E−09	7.39458E−05	35.33258204
	134,161,296	rs4434650	T	C	0.102642	0.0882	0.0169	1.892E−07	5.70043E−05	27.23716759
	28,094,919	rs562598618	A	G	0.0144969	−0.2296	0.0477	1.46599E−06	4.84902E−05	23.16888807
	17,560,917	rs72844539	A	G	0.0255884	0.1782	0.0369	1.40301E−06	4.88101E−05	23.32173462
	48,733,224	rs76103846	T	A	0.0499617	0.1404	0.0297	2.28602E−06	4.67702E−05	22.34701389
	49,120,484	rs77837771	G	C	0.132024	0.1087	0.0228	1.93001E−06	4.75704E−05	22.72937931

A = adenine, C = cytosine, EAF = effect allele frequency, G = guanine, IgAN = immunoglobulin A nephropathy, MR = Mendelian randomization, SE = standard error, SNP = Single nucleotide polymorphism, T = thymine.

**Figure 2.**
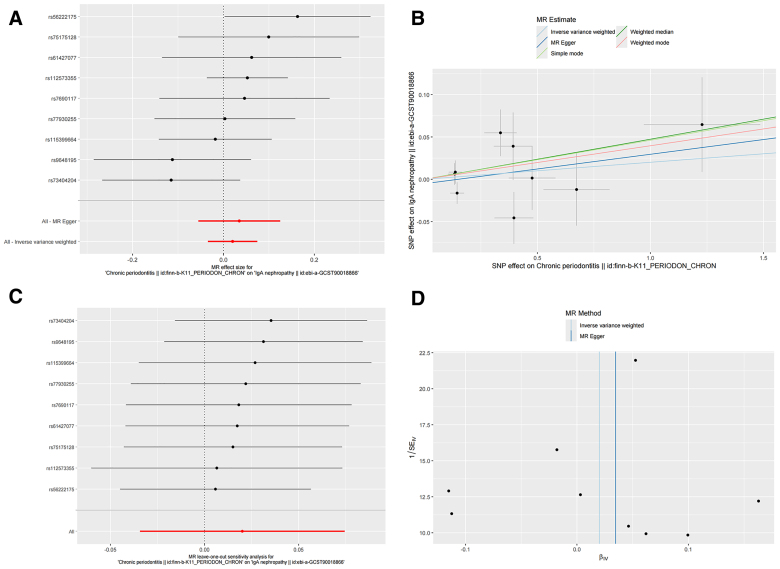
The causal effect of chronic periodontitis on IgA nephropathy. (A) Forest plot, (B) Scatter plot, (C) Leave-one-out plot, and (D) Funnel plot. IgA = immunoglobulin A, MR = Mendelian randomization, SE = standard error, SNP = single nucleotide polymorphism.

Table 2 presents the outcomes of various MR analysis methods regarding the causal effect of chronic periodontitis on IgAN. Owing to the absence of heterogeneity, as demonstrated by the Cochran *Q* test (*Q* = 10.11, *P *= .26), fixed-effect IVW models were used for the main analysis. However, no outliers were detected using the MR-PRESSO global test (RSSobs = 12.66, *P *= .28). The IVW method found no genetic evidence for a strong causal effect of chronic periodontitis on IgAN in the genetic prediction (odds ratio [OR] = 1.02, 95% CI = 0.97–1.07, *P* = .42). Similar to the IVW method, other methods produced results in which the OR value was approximately 1, and the *P* value did not reach statistical significance. Consequently, MR analysis results from databases suggest that chronic periodontitis does not increase the risk of IgAN. Figure 2A displays the forest plot of pooled MR estimates and individual estimates between chronic periodontitis-associated SNPs and the risk for IgAN. The scatter plot of the causal effect provided by each MR estimator is displayed in Figure 2B. Additionally, directional pleiotropy was not found because the intercept did not reach significance (intercept = −0.005, SE = 0.01, *P* = .69), indicating that directional pleiotropy was not observed. Figure 2C demonstrates that in the leave-one-out sensitivity analysis, no single SNP significantly influenced the overall effect of chronic periodontitis on IgAN. The Cochran *Q* test and funnel plot (Fig. 2D) indicated the absence of heterogeneity. There was a 19.73% sample overlap between individuals with chronic periodontitis and those with IgAN. The MRlap analyses were consistent with the primary results, indicating a nonsignificant difference (*P* for difference = .99). Consequently, despite the partial overlap between the 2 samples, a considerably weak instrument bias would not be expected. The total variance explained for chronic periodontitis was 0.00098, and the statistical power for detecting the causal effect of chronic periodontitis on IgAN was 0.05.

**Table 2:** Results of causal effect of chronic periodontitis on IgAN.

Exposure	Outcome	SNPs	MR method	OR (95% CI)	*P* value
Chronic periodontitis	IgAN	9	MR Egger	1.04 (0.95–1.13)	.47
			Weighted median	1.05 (0.98–1.12)	.16
			IVW	1.02 (0.97–1.07)	.42

CI = confidence interval, IgAN = immunoglobulin A nephropathy, IVW = inverse variance weighted, MR = Mendelian randomization, OR = odds ratio, SNP = single nucleotide polymorphism.

**Figure 3.**
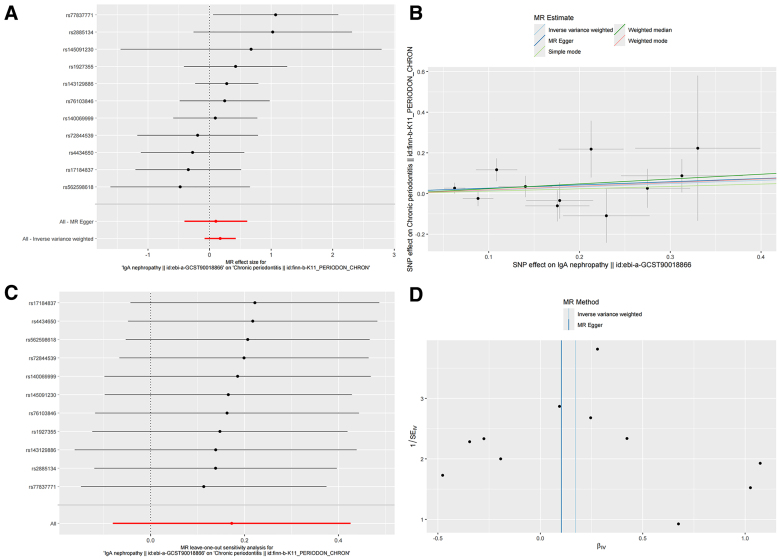
The causal effect of IgA nephropathy on chronic periodontitis. (A) Forest plot, (B) Scatter plot, (C) Leave-one-out plot, and (D) Funnel plot. IgA = immunoglobulin A, MR = Mendelian randomization, SE = standard error, SNP = single nucleotide polymorphism.

Inverse MR analyses revealed no genetic evidence for a strong causal effect of IgAN on chronic periodontitis (OR = 1.19, 95% CI: 0.92–1.53, *P* = .18). The results of MR-Egger and weighted median analyses were consistent with those of the IVW analysis (Table 3). The Cochran *Q* test did not find any major heterogeneity that would affect the evaluation of causality (*Q* = 9.81, *P* = .46). Horizontal pleiotropy was not detected using the MR-Egger intercept test (intercept = 0.01, SE = 0.03, *P* = .76). No specific outliers were identified using the MR-PRESSO global test (RSSobs = 12.69, *P *= .48). Figure 3A displays the forest plot of pooled MR estimates and individual estimates between IgAN-associated SNPs and the risk of chronic periodontitis. Figure 3B displays the scatter plot of the causal effect given by each MR estimator. The leave-one-out test confirmed that no causal association was affected by a specific SNP (Fig. 3C). The Cochran *Q* test and funnel plot (Fig. 3D) indicated no heterogeneity. Given the focused hypothesis, multiple tests for bidirectional analyses were not performed. There was a 67.23% sample overlap between the individuals with IgAN and those with chronic periodontitis. The MRlap analyses were consistent with the primary results, indicating a nonsignificant difference (*P* for difference = .25). Consequently, despite the partial overlap between the 2 samples, a significant weak instrument bias was not anticipated. The total variance explained for chronic periodontitis was 0.00060, and the statistical power for detecting the causal effect of IgAN on chronic periodontitis was 0.09.

**Table 3:** Results of causal effect of IgAN on chronic periodontitis.

Exposure	Outcome	SNPs	MR method	OR (95% CI)	*P* value
IgAN	Chronic periodontitis	11	MR Egger	1.11 (0.67–1.84)	.7
			Weighted median	1.27 (0.9–1.81)	.18
			IVW	1.19 (0.92–1.53)	.18

CI = confidence interval, IgAN = immunoglobulin A nephropathy, IVW = inverse variance weighted, MR = Mendelian randomization, OR = odds ratio, SNP = single nucleotide polymorphism.

## 4. Discussion

This study is the first to comprehensively explore the genetic association between chronic periodontitis and IgAN using GWAS data. Previous epidemiological studies mostly used case-control or cross-sectional designs, which were unable to directly identify causal relationships between the variables. Even in prospective observational studies, interfering confounding risk factors are difficult to avoid. Using MR methods in this study, we identified causal relationships that extended beyond confounding factors. According to IVW estimates, we found no genetic evidence for a strong causal effect between chronic periodontitis and IgAN.

Several studies have suggested that IgAN is more common in patients with chronic periodontitis than in the general population.^[18]^ This MR study found no genetic evidence for a strong causal effect between chronic periodontitis and IgAN. There are various explanations for the discrepancies between our results and those of previous studies.

Multiple studies have confirmed the clinical association between chronic periodontitis and IgAN. According to some studies, clinical remission of IgAN is positively impacted by periodontal therapy,^[32,33]^ but there is no direct causal relationship between them. In chronic periodontitis, they are connected indirectly through the colonization, metabolism, and immune activation of specific pathogenic bacteria.^[17,18]^ These bacteria contribute to the onset and progression of IgAN by controlling immunological imbalance, increasing oxidative stress, and prompting abnormal IgA1 production, these bacteria contribute to the onset and progression of IgAN production.^[34,35]^ Several anaerobic bacterial genera implicated in periodontitis have also been associated with IgAN.^[36–38]^ The pathogenic bacterial community associated with chronic periodontitis is complex, and research has confirmed that *Treponema denticola*, *Porphyromonas gingivalis,* and *Campylobacter rectus* are associated with the incidence and pathogenesis of IgAN.^[14,39]^ *P. gingivalis* in the oral cavity contributes to the pathogenesis of IgAN by inducing elevated levels of galactose-deficient immunoglobulin A1 (Gd-IgA1). The oral mucosal barrier is disrupted, and local chronic inflammation is induced by virulence factors such as gingipains. The lipopolysaccharide of *P. gingivalis* can simultaneously trigger systemic immune responses, promote helper T cell 17 differentiation, and inhibit the function of regulatory T cells, leading to helper T cell 17/regulatory T cells imbalance and further enhancing Gd-IgA1 production. Gd-IgA1, the core pathogenic factor of IgAN, forms immune complexes that deposit in the glomerular mesangium.^[40]^ Animal model experiments have confirmed that intranasal administration of *P. gingivalis* in mice leads to mesangial proliferation and IgA deposition.^[14]^ These results suggest that *P, gingivalis* plays a role in IgAN pathogenesis. These findings suggest that the occurrence of IgAN is associated with specific bacterial communities that lead to chronic periodontitis.

Individuals’ long-term exposure to periodontal-associated pathogenic microorganisms and the chronic inflammatory state they induce can be reliably predicted by genetic variations closely associated with chronic periodontitis. The lifelong cumulative effects of specific microbial/inflammatory exposures on IgAN, which is represented by chronic periodontitis, can be effectively simulated using these genetic variations as IVs because they are identified at birth and are independent of environmental and behavioral factors. This method helps circumvent the confounding bias and reverse causality commonly encountered in traditional observational studies, thereby providing more reliable genetic evidence for evaluating potential causal associations between exposure and outcomes. Access to a substantial sample of GWAS summary data for IgAN and chronic periodontitis is one of the main advantages. Additionally, using SNPs as IVs can avoid reverse causality and confounding factors, which is an inherent advantage of MR analysis. All samples were sourced from the European population to avoid bias due to racial differences.

However, this study has certain limitations. First, all GWAS data were obtained from European populations. Whether the results of the studies discussed apply to other populations remains to be determined. One limitation of our study is the lack of individual-level data, which hinders our ability to utilize longitudinal data to estimate the causal effect of chronic periodontitis on IgAN. Additionally, small effect sizes, nonlinear effects, or residual confounding might go unnoticed. MR estimates may not adequately account for environmental or pathogen-driven diseases because they reflect lifelong genetic susceptibility to periodontitis. Moreover, given the null findings, it is important to consider the statistical power of the present MR analysis. These low power estimates suggest that the current study can only confidently exclude strong causal effects, whereas small to moderate effects remain possible. Accordingly, the null findings should be interpreted with caution, and larger-scale GWAS datasets are warranted to achieve sufficient power to detect modest causal associations.

## 5. Conclusion

In this MR analysis, we found no genetic evidence for a strong causal effect of chronic periodontitis on IgAN, or vice versa, in the European population. However, high-quality, multicenter, and prospective randomized controlled trials are warranted to provide more definitive evidence on this issue. Future studies on the molecular mechanisms underlying chronic periodontitis and IgAN will be conducted as more comprehensive GWAS data become available.

## Author contributions

**Conceptualization:** Runmiao Hua.

**Data curation:** He Miao.

**Formal analysis:** He Miao.

**Methodology:** Runmiao Hua.

**Software:** He Miao, Jiaguo Huang.

**Validation:** Jiaguo Huang.

**Writing** – **original draft:** He Miao, Feifei Guan.

**Writing** – **review & editing:** Jiaguo Huang, Runmiao Hua.
